# Design and Implementation of a Genomics Field Trip Program Aimed at Secondary School Students

**DOI:** 10.1371/journal.pcbi.1002636

**Published:** 2012-08-30

**Authors:** Jennifer McQueen, Jody J. Wright, Joanne A. Fox

**Affiliations:** 1 Genetics Graduate Program, University of British Columbia, Vancouver, British Columbia, Canada; 2 Department of Microbiology & Immunology, University of British Columbia, Vancouver, British Columbia, Canada; 3 Michael Smith Laboratories, University of British Columbia, Vancouver, British Columbia, Canada; Whitehead Institute, United States of America

## Abstract

With the rapid pace of advancements in biological research brought about by the application of computer science and information technology, we believe the time is right for introducing genomics and bioinformatics tools and concepts to secondary school students. Our approach has been to offer a full-day field trip in our research facility where secondary school students carry out experiments at the laboratory bench and on a laptop computer. This experience offers benefits for students, teachers, and field trip instructors. In delivering a wide variety of science outreach and education programs, we have learned that a number of factors contribute to designing a successful experience for secondary school students. First, it is important to engage students with authentic and fun activities that are linked to real-world applications and/or research questions. Second, connecting with a local high school teacher to pilot programs and linking to curricula taught in secondary schools will enrich the field trip experience. Whether or not programs are linked directly to local teachers, it is important to be flexible and build in mechanisms for collecting feedback in field trip programs. Finally, graduate students can be very powerful mentors for students and should be encouraged to share their enthusiasm for science and to talk about career paths. Our experiences suggest a real need for effective science outreach programs at the secondary school level and that genomics and bioinformatics are ideal areas to explore.

## Introduction

Genomics technologies and bioinformatics analyses have changed the way that biological research is being carried out today. Given the rapid pace of advancements in these fields, the development of science-literate citizens can be greatly aided by introducing current scientific breakthroughs and technologies early in high school curricula. Genomics is an ideally suited subject area for designing and implementing high school education initiatives because of its broad relevance to many fields of cutting-edge research, the ability to engage students with freely available online tools, as well as the availability of a growing number of teaching and curricula resources [1]. Experiential learning activities, such as field trips, have been shown to be extremely effective for shifting student beliefs about science [2], and we agree with those who have argued that the time is right for developing genomics and bioinformatics activities for secondary school students [1]. It is crucial for youth to have a basic understanding of modern life sciences research including human genetics and genomics as current generations will be faced with challenging personal decisions regarding whether or not to have their genomes sequenced, who has the right to access their genetic information, and how that information might be used [3]. In addition to educating students so that they can navigate public debate around these issues, education efforts can have long-term benefits by ensuring that science is an attractive career choice for young people [4]. Many teachers and researchers have also recognized that bioinformatics can be a valuable tool for engaging students with many core biological concepts covered in high school while at the same time touching on themes of mathematics, computer science, and technology [1], [4], [5]. Several broad strategies for strengthening secondary school education efforts in the sciences have been suggested including support for teacher training and/or efforts to attract students into pursuing further studies and careers in science, technology, engineering, and mathematics [6]. While others have successfully focused on developing bioinformatics training for high school teachers [5], our approach has been to provide a full-day genomics and introductory bioinformatics field trip program for secondary school students. Using active learning strategies and hands-on activities to engage students, we aim to complement curricula covered in high school classrooms, generate an interest in the sciences, and provide mentorship for career choices in the sciences.

Our Genomics Field Trip Program offers secondary school students, teachers, and parent chaperones an integrated laboratory and computer-based hands-on experience hosted in the Michael Smith Laboratories (MSL) at the University of British Columbia (UBC). Named after Michael Smith (1993 Nobel Prize winner in Chemistry), the MSL houses research laboratories, educational facilities, and space open to the public, and is home to research groups from 10 different departments at UBC. We have commented previously on this unique atmosphere and the key advantages that this crucial link to university research offers for designing impactful education experiences [7], [8]. The Genomics Field Trip Program described here operates alongside several different science outreach programs offered at the educational facilities of the MSL (http://bioteach.ubc.ca) that reach out to elementary-, junior-, and senior-level students, high school teachers, and the general public.

In this article, we describe the objectives, content, and logistics of the Genomics Field Trip Program hosted at the MSL; we discuss the benefits of research-based field trip programs for students, facilitators, and teachers; and we present a list of recommendations for coordinating and facilitating effective high school field trip programs within research facilities.

## A Day in the Genomics Field Trip Program

The Genomics Field Trip Program is a full-day (5 h) high school field trip hosted at the MSL at UBC. UBC's Faculty of Science is a world-class research faculty that educates 7,000 undergraduate students and 1,300 graduate students per year and engages with communities through museums and outreach programs including those hosted at the MSL. Teachers bring their classes (typically 24–32 students) to visit our research facility and participate in an authentic laboratory experience designed for Grade 9 students (14–15-year-olds). Over the past 3 years, more than 1,300 students and teachers have experienced our full-day genomics field trip that explores concepts related to DNA, DNA sequencing, genomes, and personal genomics. The flow of experiments, demonstrations, and activities covered in this field trip is outlined in Table 1. Our laboratory space is organized with 15 laptops placed directly on the laboratory research benches so that pairs of students can work together to complete the fully integrated set of wet-lab and dry-lab activities. After isolating their own DNA, students participate in an inquiry-based activity using the computers to research the definition of “genome” and “genome sequencing,” as well as learn more about the human genome sequencing project. Students then explore the relationship between DNA sequence and protein in a paper-and-pen-based bioinformatics activity where they align short overlapping DNA sequence reads and then transcribe and translate the full-length DNA sequence. Our field trip curriculum encourages students to extend their own understanding of fundamental concepts taught in their classrooms and textbooks, for example, by exploring the role of the environment (toxins, social interactions, exercise, diet) on gene expression. Finally, students address the impacts of genetic knowledge on society by discussing several controversies related to human genome sequencing and personal genomics. A resource page with example lesson plans, more information about key concepts covered, and all activities is available online at http://www.bioteach.ubc.ca/genetics-fieldtrips/.

**Table 1:** Topics and activities covered by the Genomics Field Trip Program.

Section	min	Topics Covered	Activities
**Welcome**	15	Applications of Research in Genomics and Bioinformatics	1. Graduate student instructors discuss their own research projects/interests
		Laboratory Safety	2. Orientation to building
			3. Safety and rules
**Properties of DNA**	60	**“DNA Isolation”**	4. Presentation/discuss components of DNA and DNA extraction
		Physical and Chemical Properties of DNA	5. Students extract DNA from cheek cells^a^
		DNA Extraction	6. Students recognize a DNA precipitate
			7. Students label DNA drawing and list reagents used in DNA extraction
**Break**	15		8. Michael Smith - Nobel laureate quiz
**Information in DNA and Genomes**	60	**“Transcription/Translation”**	9. Students perform online searches to define genome, sequencing, and the Human Genome Project (HGP)
		Genomics and the Human Genome Project	10. Class discuss answers to HGP definitions
		Relationship between Genes and Proteins	11. Students watch video about the HGP
		Relationship between Genomes, Genes, and Human Traits	12. As groups of 4–6, students race to put in order overlapping short DNA sequences
			13. Class discuss and watch video about transcription and translation
			14. Students transcribe and translate the short DNA sequence
			15. Presentation and questions regarding the role of environment and genotype of phenotype
**Lunch**	45		
**Graduate Student Q&A**	15	University Life	16. Students write questions about university life or careers in science that graduate student instructors answer
		Careers in Science	
**Human Genomic Controversies**	45	**“Genomic Controversies”**	17. Watch video that introduces controversies within the field of human genomics
		Impacts of Genetic Knowledge on Society	18. Students read through personal genomics controversies worksheet
		Personal Genomics	19. Answer personal genomics questions in groups 4–6
		Pre-Implantation Diagnosis	20. Students watch video regarding human reproduction (pre-implantation diagnosis)
			21. Read through handout and discuss pre-implantation diagnosis questions in small groups
**Summary**	30	**“Jeopardy Game”**	22. Students play a game of Jeopardy that reviews material covered in class
		Summary of Key Concepts	
**Feedback**	15		23. Each student fills out an online feedback form
			24. High school teacher fills out a paper feedback form

a *Cracking the Code of Life-Teacher's Guide*: http://www.pbs.org/wgbh/nova/teachers/activities/2809_genome.html.

Our program was developed in consultation with high school teachers and aligns directly with curricula guidelines and learning goals set by our Provincial Government. Each section of the field trip has its own set of learning objectives and hands-on activities (such as performing a DNA isolation) that accompany each objective (Table 1 and Table S1). This program specifically targets a youth audience (14–16-year-olds) with the aim of making a difference in young students' attitudes towards science early enough to influence decisions about senior-level science classes. The first version of this field trip was piloted with eight different classroom visits in the Fall of 2009. In each of these pilot sessions (and in every session since), we surveyed teachers regarding the effectiveness of each activity. These pilot sessions were essential and teachers provided invaluable suggestions that guided activity and resource development. For example, teachers suggested that worksheets would help their classes stay focused and requested pre–field trip information containing vocabulary lists (see Text S1).

## Logistics

**Figure 1:**
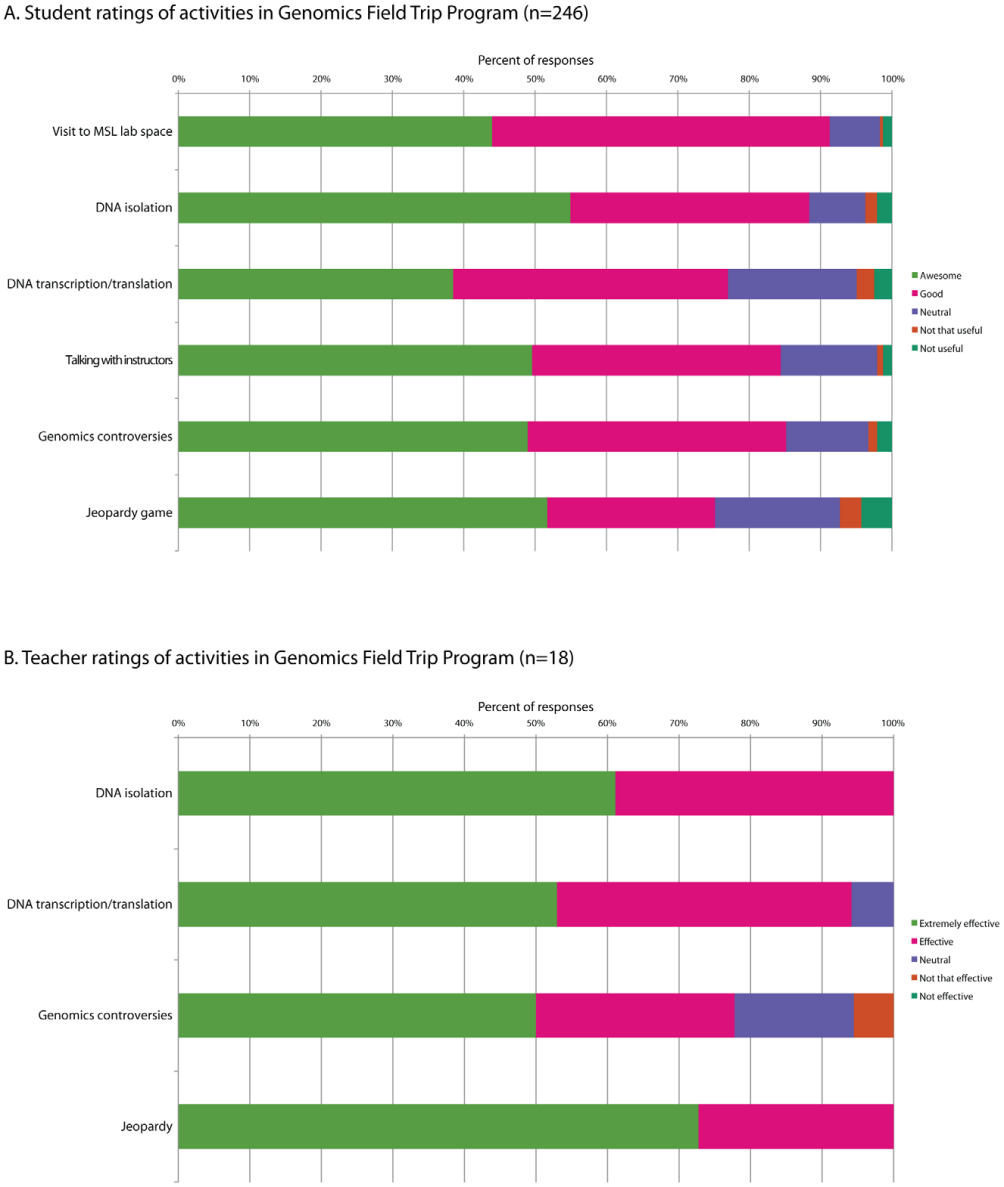
Ratings of field trip activities by students and teachers. (A) Student and (B) teacher ratings of activities in Genomics Field Trip Program sessions occurring between September 2011 and February 2012 (*n* = 246 and 18, respectively, from 14 different classroom visits).

The Genomics Field Trip Program has been running at the MSL for 3 years (2009–2012) with classroom visits scheduled between October and May and one Grade 9 class visiting each week. No pre-requisites are required for groups to attend. Teachers are provided with a pre-reading document in advance that includes topics, terminology, and learning objectives they may wish to address in their classrooms prior to attending. The workshops are offered free of charge based on support from the MSL and a grant awarded by the PromoScience Program of the Natural Sciences and Engineering Research Council of Canada (NSERC). High school classes are accompanied by one or more teachers or parent chaperones, and field trips are guided by two instructors. The instructors typically consist of graduate students drawn from various graduate programs at UBC, including Genetics, Microbiology, Bioinformatics, and Genome Science & Technology. Hosts are selected based on enthusiasm for teaching and familiarity with the fields of genomics and bioinformatics. Host training programs are provided in September each year in order to review the learning objectives, activities, and logistics of the field trips in addition to providing guidelines for interacting with high school students effectively. In order to run weekly programs, 4–5 instructors rotate hosting duties throughout the year. Hosts in training observe at least one field trip session led by experienced hosts and also co-teach with an experienced host for their first one to two sessions, or until they are comfortable leading the program. With our diverse set of science outreach programs at the MSL, we have developed a network of 300+ science high school teachers across BC, and advertise opportunities through our website (http://www.bioteach.ubc.ca) and through word-of-mouth recommendations only. We are fortunate to have excellent designated space for our activities, including a modern laboratory (capable of holding 32 students), a lecture hall, and a multipurpose space. In order to evaluate the effectiveness of our field trip program, each student answers an online survey relating to the usefulness of the activities, the effectiveness of interactions with program instructors, as well as their perspectives on science and engineering as future areas of study or vocation (Figure 1 and Text S1). Teachers are also surveyed regarding the usefulness of field trip activities (Figure 1) and the effectiveness of the field trip program in assisting in the delivery of specific curricula as outlined by the Provincial Government (unpublished data).

## Benefits

We believe that delivering science outreach programs that relate to facilitators' research interests offers benefits to learners, teachers, and facilitators alike [7]. The students who attend our program have varying levels of interest in the sciences, technology, engineering, and math. We focus on using the topic areas of bioinformatics and personal genomics to make connections to these students' own lives as well as to their personal interests. The student evaluation data collected over the 3 years of this project show that students who have participated are more interested in the sciences and engineering, with less than 5% of students indicating that they are not interested in the sciences and more than 40% responding that they are “unsure” about their studies and/or careers (*n* = 951, student responses collected between May 2009 and January 2012). This large percentage of students who are not sure about their future in science speaks directly to the need for programs like ours that engage and inspire students with science in fun ways that are relevant to their own lives. All students surveyed responded positively to this field trip experience in open-ended questions on our survey. For example, a student participant comments, “This program allows us to see how useful science contributes to our everyday life.” Our program directly addresses this issue with a question-and-answer session with graduate student mentors about university life and career options in the sciences (Table 1). Another student writes, “We learned that science isn't just spending time in the labs, wearing coats. There are lots of different jobs we can go into if we continue studying science.” High school teachers place a high value on these interactions with our field trips instructors, who provide valuable role models for career paths and post-secondary choices in the sciences.

Our program instructors are typically graduate students who can also benefit from the unique teaching experiences that the high school field trips offer. Firstly, field trip instructors develop their ability to communicate scientific ideas to a general public, a skill greatly needed within our scientific community. Secondly, instructors gain experiences in lesson design, lesson delivery, and the incorporation of feedback into future lessons. This chance to build teaching experiences and to enhance their instructional skills does not always occur in standard teaching assistantship positions. Lastly, there is great personal satisfaction gained from sharing a passion for the sciences with others.

## Recommendations for Design and Implementation of Field Trip Programs

Through our involvement with the Genomics Field Trip Program and other science outreach field trips hosted at the MSL over the past 3 years, we have learned a lot about designing and implementing secondary school field trip programs within existing research facilities (see Box 1). We encourage you to pilot similar programs at your institution and recommend that you first check your content against each of the 10 simple rules for teaching bioinformatics at the high school level put forward by Form and Lewitter [9]. Our lessons learned (see Box 1) reinforce these rules and offer additional advice about leveraging the unique onsite experience that a field trip offers.

**Box 1. Lessons Learned from Designing & Implementing Secondary School Field Trips**

***1. Be yourself.***

Create a field trip program based on content you are actually interested in. This makes it much easier to get excited and thus to get participants excited about science. One of the biggest rewards associated with doing outreach as a scientist is getting the opportunity to share your work and your excitement about it with the public—including the next generation of potential researchers.

***2. Tie concepts to applications and examples with which students are familiar.***

There are numerous themes in popular culture and the media relating to genomics and computational biology, for example, in Hollywood films such as GATTACA, X-men, and Contagion, television shows such as CSI, and in the application of therapies such as gene doping in high performance sports. Build on the exposure to science participants already have by making explicit connections between material learned in the field trip and examples found in popular culture and daily life.

***3. Link learning objectives to existing secondary school curricula.***

Run a pilot program with several local teachers and their classes who have indicated a willingness to provide feedback to assist connecting field trip objectives and content to regional curricula.

Provide teachers with a pre-reading document in advance which includes topics, terminology, and learning objectives they may wish to address in their classrooms prior to attending.

***4. Focus on having fun.***

While making sure field trip programs are relevant, interesting, and tied to local curricula is important, making sure programs are fun is of paramount importance for ensuring long term success. Choosing facilitators who are passionate about the material makes it easier to focus on fun, as does incorporating a wide variety of activities and games into the day, such as Jeopardy, pop quizzes, races to complete class activities, and scavenger hunts.

***5. Create dedicated time in the day for participants to ask questions about university life, research, and careers in science and engineering.***

The majority of students polled in the Genomics Field Trip Program reported that they are unsure about pursuing education and/or careers in the sciences and engineering. This may partly be due to misunderstanding or uncertainty about what such educational opportunities or careers might actually look like. We think that scientists and trainees can offer valuable advice and address this uncertainty by giving students opportunities to ask about university life and careers in the sciences in addition to providing examples which highlight the vast diversity of careers available in the sciences and engineering.

***6. Create opportunities for graduate and senior undergraduate students to participate in organizing and teaching the field trips.***

Many graduate and senior undergraduate students are keen to find additional ways to practice and improve their teaching skills. A field trip program provides excellent opportunities to practice teaching in a low-risk environment as well as opportunities to engage in curriculum review and development.

Graduate students can be very powerful mentors by sharing their own career paths and their enthusiasm for science.

***7. Use surveys to evaluate effectiveness of programs and make curriculum changes accordingly.***

A simple online or paper survey is easy to perform and has high returns for ensuring program success. Surveys provide invaluable information about the effectiveness of certain activities in achieving stated learning goals. Build program review (including review of instructor perceptions as well as teacher and participant surveys) into the annual management tasks for the program and adjust the program accordingly each term or each year.

## Future Directions

While the current format of the Genomics Field Trip Program appears to be enjoyable and effective based on the results of teacher and student surveys, we are eager to continue improving the program and our ability to assess its impact. In future years, we plan to incorporate pre–field trip evaluations in order to better assess the effectiveness of our program at assisting students in achieving desired learning goals and to measure changes in student attitudes. We plan to extend the DNA transcription and translation activity from a paper-and-pen-based activity into introductory bioinformatics activities performed using public freely available software tools on our laptops. We also aim to incorporate activities that explore the impact of mutations in DNA sequence on protein structure and function, using interactive 3D protein structure visualization tools and tactile models.

## Conclusion

The exciting fields of genomics and bioinformatics are ideally suited to help secondary school students understand modern life sciences and to make connections between biological science and their daily lives. Designing and implementing genomics field trip programs is relatively simple, immensely rewarding, and provides ample benefits for all parties involved. Engaging with local teachers to build on regional high school curricula as well as learning from existing field trip programs are both effective strategies for ensuring program success. High school field trip programs hosted at research facilities hold great potential for connecting university research to the community at large and have a positive influence on student perceptions and interest in science as a field of study or vocation.

## Supporting Information

Table S1 Key concepts addressed by activities in Genomics Field Trip Program.
(PDF)

Text S1 Resource package for Genomics Field Trip Program. This resource package includes all lesson plans, worksheets, answer keys, and teacher resources associated with the Genomics Field Trip Program. Student and teacher evaluation forms are also included. All of these materials are also available on our field trip resource page, http://www.bioteach.ubc.ca/genetics-fieldtrips/.
(PDF)
