# Supplementary material for: Design and Implementation of a Genomics Field Trip Program Aimed at Secondary School Students
Source: PLoS Comput Biol. 2012 Aug 30;8(8):e1002636. doi: 10.1371/journal.pcbi.1002636 (PMC3431290; doi:10.1371/journal.pcbi.1002636)
Supplement: Table S1 — Key concepts addressed by activities in Genomics Field Trip Program. (PDF) [file pcbi.1002636.s001.pdf]

Table S1. Key Concepts Addressed by Activities in Genomics Field Trip Program.

Category	Key Concepts[1,2]	Activities
Process of Science	• Demonstrate safe laboratory procedures and behavior.<br>• Perform experiments using the scientific method.<br>• Demonstrate scientific literacy.<br>• Demonstrate ethical, responsible, co-operative behavior.<br>• Describe the relationship between scientific principles and technology.	1. Graduate student instructors discuss their own research projects/interests<br>2. Orientation to building<br>3. Safety and rules<br>5. Students extract DNA from cheek cells<br>6. Students recognize a DNA precipitate<br>8. Michael Smith - Nobel laureate quiz<br>16. Students write questions about university life or careers in science that graduate student instructors answer
Nature of Genetic Material; Reproduction	• DNA is the genetic material for all living organisms.<br>• Genes are segments of DNA that encode information critical for development. DNA is organized into structures called chromosomes.<br>• Identify chromosomes, nucleotides, base pairs, sugar-phosphate backbone, double helix, centromere, nucleus, cell membrane, nuclear membrane.<br>• The genome is all the genetic information within an organism.<br>• The amount of genetic information within an organism is not a product of size or complexity.<br>• Genome sequencing involves producing and organizing short overlapping DNA sequences.<br>• Humans receive half their genetic information from each parent through the processes of replication, meiosis and fertilization.<br>• Relate the processes of cell division and emerging reproductive technologies to embryonic development.	4. Presentation/ Discuss components of DNA and DNA extraction<br>5. Students extract DNA from cheek cells<br>6. Students recognize a DNA precipitate<br>7. Students label DNA drawing and list reagents used in DNA extraction<br>9. Students perform online searches to define genome, sequencing and the Human Genome Project (HGP)<br>10. Class discuss answers to HGP definitions<br>11. Students watch video about the HGP<br>12. As groups of 4-6, students race to put in order overlapping short DNA sequences<br>17. Watch video that introduces controversies within the field of human genomics<br>18. Students read through personal genomics controversies worksheet<br>19. Answer personal genomics questions in groups 4-6<br>20. Students watch video regarding human reproduction (pre-implantation diagnosis)<br>21. Read through hand out and discuss pre-implantation diagnosis questions in small groups
Gene Expression and Regulation	• Genes exist in different forms called alleles.<br>• The expression of genetic information generally flows from DNA to RNA to protein. This occurs through transcription of DNA into RNA and translation of mRNA into protein.<br>• Virtually all cells within a human body contain the same genetic information.<br>• For traits primarily influenced by single genes, certain combinations of alleles lead to predictable patterns of inheritance. Other more complex traits involve the influence of multiple genes.<br>• The functions of genes and their products can be affected by the environment and other genes at one or many steps involved in producing a trait.	9. Students perform online searches to define genome, sequencing and the HGP<br>10. Class discuss answers to HGP definitions<br>11. Students watch video about the HGP<br>12. As groups of 4-6, students race to put in order overlapping short DNA sequences<br>13. Class discuss and watch video about transcription and translation<br>14. Students transcribe and translate the short DNA sequence<br>15. Presentation and questions regarding the role of environment and genotype of phenotype

Key Concepts adapted from:
[1] B.C. Ministry of Education. Grade 9 Curriculum Package, online. www.bced.gov.bc.ca/irp [April 11, 2012]
[2] Dougherty M, Pleasants C, Solow L, Wong A, et al. (2011) A Comprehensive analysis of High School Genetics Standards: Are States Keeping Pace with Modern Genetics? CBE- Life Sciences Education (10): 318-327.
