# Supplementary material for: Design and Implementation of a Genomics Field Trip Program Aimed at Secondary School Students
Source: PLoS Comput Biol. 2012 Aug 30;8(8):e1002636. doi: 10.1371/journal.pcbi.1002636 (PMC3431290; doi:10.1371/journal.pcbi.1002636)
Supplement: Text S1 — Resource package for Genomics Field Trip Program. This resource package includes all lesson plans, worksheets, answer keys, and teacher resources associated with the Genomics Field Trip Program. Student and teacher evaluation forms are also included. All of these materials are also available on our field trip resource page, http://www.bioteach.ubc.ca/genetics-fieldtrips/. (PDF) [file pcbi.1002636.s002.pdf]

Lesson Plan: Properties of DNA

Key Concepts Covered[1,2]

PROCESS OF SCIENCE:
- Demonstrate safe laboratory procedures and behavior
- Perform experiments using the scientific method

NATURE OF GENETIC MATERIAL; REPRODUCTION:
- DNA is the genetic material for all living organisms.
- Genes are segments of DNA that encode information critical for development. DNA is organized into structures called chromosomes.
- Identify chromosomes, nucleotides, base pairs, sugar-phosphate backbone, double helix, centromere, nucleus, cell membrane, nuclear membrane.
- Humans receive half their genetic information from each parent through the processes of replication, meiosis and fertilization.

Overview:
This 60 min lesson plan covers 6 components of DNA as well as a general protocol to extract DNA. Students use this knowledge to extract DNA from their own cheek cells. Learning is solidified by labeling a DNA diagram and writing down the reagents used in the DNA extraction in a handout. This handout is used for subsequent sections of the MSL Genomics Field Trip and is available as an adjoining PDF (MSL OUTREACH GENOMICS worksheet).

Learning Objectives:
By the end of this lesson, students will be able to identify components of DNA and list properties of DNA as well as recognize a DNA precipitate in a simple genomic extraction.

Resources Needed:
For each DNA extraction (one per student): 5 mL 0.9% Saline solution, 0.5 mL 10% SDS, 10mL 100% Ethanol, 1X Dixie Cup, 1X Microcentrifuge tube)
Handout, 1 printout per student (see attached PDF, MSL OUTREACH GENOMICS worksheet)

Introduction/ Hook:
- Why are we going to spend a day talking about DNA? Why do you think your teacher thought it was important to bring you here?
- Your lives will take you many places but I would like to convince you that DNA is important to many of your interest and will affect your lives in many ways.

Presentation explaining how DNA impacts various aspects of lives (Human behavior, health, law and justice, human history, biodiversity and the environment, energy production, arts)

Pre-Assessment: While displaying an image of DNA have students identify parts familiar to them (Double helix, sugar-phosphate backbone, nucleic acids, chromosomes, centromere, base pairs). Discuss the unfamiliar and if all familiar talk in more detail about each component.
- If we want to explore what DNA is we are going to have to look at it. Any thoughts on how we could extract DNA?

Teacher Resources for DNA Isolation Activity:
[1]Cracking the Code of Life- Teacher's Guide: http://www.pbs.org/wgbh/nova/teachers/activities/2809_genome.html

Activity: DNA Isolation
Explain the steps of a DNA extraction. Handout supplies needed for DNA extraction. As a group go through the steps in DNA extraction.

1. **Cell collection:** Swish 5 mL saline solution in mouth for 30 seconds, spit into Dixie cup, pour spit back into saline container.
2. **Break up membranes:** Add 0.5 mL SDS and wait 5 minutes.
3. **Precipitate DNA out of solution:** Pour 10 mL 100% Ethanol slowly into container, so as not to mix up the two layers. Watch the interface between the two layers for the precipitate of DNA.
4. **Transfer DNA precipitate** into microcentrifuge tube.

Post- test/ Summary Have students fill out the handout, labeling the 6 components of DNA mentioned and the 3 reagents used in the DNA extraction. Have them double check their answers with their neighbors then go over answers as a class.

Key Concepts adapted from:
[1] B.C. Ministry of Education. Grade 9 Curriculum Package, online. www.bced.gov.bc.ca/irp [April 11, 2012]
[2] Dougherty M, Pleasants C, Solow L, Wong A, et al. (2011) A Comprehensive analysis of High School Genetics Standards: Are States Keeping Pace with Modern Genetics? CBE- Life Sciences Education (10): 318-327.

Credits: This lesson plan was developed by Jennifer McQueen, Jody Wright, and Joanne Fox as part of the science outreach efforts at the Michael Smith Laboratories at the University of British Columbia, http://bioteach.ubc.ca

Lesson Plan: The Information in DNA

Key Concepts Covered[1,2]

NATURE OF GENETIC MATERIAL; REPRODUCTION:

- DNA is the genetic material for all living organisms.
- Genes are segments of DNA that encode information critical for development. DNA is organized into structures called chromosomes.
- The genome is all the genetic information within an organism.
- The amount of genetic information within an organism is not a product of size or complexity.
- Genome sequencing involves producing and organizing short overlapping DNA sequences.

GENE EXPRESSION AND REGULATION:

- Genes exist in different forms called alleles.
- The expression of genetic information generally flows from DNA to RNA to protein. This occurs through transcription of DNA into RNA and translation of mRNA into protein.
- Virtually all cells within a human body contain the same genetic information.
- For traits primarily influenced by single genes, certain combinations of alleles lead to predictable patterns of inheritance. Other more complex traits involve the influence of multiple genes.
- The functions of genes and their products can be affected by the environment and other genes at one or many steps involved in producing a trait.

Overview:

This 60 min lesson plan covers what DNA means to organisms. It starts with definitions of several terms and then uses the story of the sequencing of the human genome project to introduce Genomics as the study of the entire genome (all of the DNA) in an organism. We make use of the competition between the public and private groups who were sequencing the human genome, to grab student's attention as they race to put in order (or align) their own overlapping sequence reads. After completing the paper DNA alignment, our attention turns to the central dogma of biology (DNA->RNA->PROTEINS). We use several learning strategies to help students understand the important processes of transcription and translation. Students watch an animation of the processes, discuss the processes, and then complete a transcription and translation exercise for the sequence they have just aligned. We end with a presentation on the effects of genotype and environment on phenotype.

Learning Objectives:

By the end of this lesson, students will be able to:

- order overlapping DNA sequence reads and merge these reads into one aligned sequence.
- transcribe and translate a DNA sequence into a protein sequence.

Resources Needed:

Laptop/ projector for watching animation
Photocopied DNA sequences
Handout, 1 printout per student (see attached PDF, MSL OUTREACH GENOMICS worksheet)

Introduction/Pre-Assessment:
Now that you can isolate DNA, what could you do with it now?

Activity: Genome Sequencing- HGP

- Students use the laptops provided to look up the terms; genome, DNA sequencing and to answer the question "Has the human genome been sequenced?" They write their answers in their own words in their handout. We go over answers as a class and probe further by asking and explaining why sequencing more human genomes is important.
- Watch short video about the HGP. http://www.genome.gov/25019885 (Introduction).
- Distribute a set of 6 short overlapping sequence reads to each group of 4-6 students. Students compete against each other to get the sequences in order. The DNA sequences are at the end of this document. We wait for and congratulate each team for finishing.

Activity: Transcription and translation

- Show image of DNA, RNA and Proteins. Introduce the central dogma concept.
- Watch video: http://www.youtube.com/watch?v=41_Ne5mS2ls (Transcription and Translation from the PBS production DNA: Secret of Life)
- Students will now transcribe and translate the sequence order in the above activity. Demonstrate with the first two codons as a group. Students use their handout to write out their own transcription and translation sequences. The handout contains the codon table.

Presentation/ Discussion: Genotype/ Environment and Phenotype
Explain terms genotype and phenotype. Ask for examples of human phenotypes. Discuss single gene traits as well as complex traits that may be influenced by more than one gene. Explain that eye colour is determined genetically, while height is dependent on other factors aside from genetics. Can they think of what this can be? Toxins! Nutrition! Introduce the concept of environment. Re-iterate that most phenotypes are due to both the environment and genetics.

Post- test/ Summary Students come up with one question they have regarding anything covered so far. They discuss their question with their group. Unanswered questions are asked to the group at large, and we discuss answers.

Key Concepts adapted from:
[1] B.C. Ministry of Education. Grade 9 Curriculum Package, online. www.bced.gov.bc.ca/irp [April 11, 2012]
[2] Dougherty M, Pleasants C, Solow L, Wong A, et al. (2011) A Comprehensive analysis of High School Genetics Standards: Are States Keeping Pace with Modern Genetics? CBE- Life Sciences Education (10): 318-327.

Credits: This lesson plan was developed by Jennifer McQueen, Jody Wright, and Joanne Fox as part of the science outreach efforts at the Michael Smith Laboratories at the University of British Columbia, http://bioteach.ubc.ca

Lesson Plan: Human Genomic Controversies

Key Concepts Covered[1,2]

NATURE OF GENETIC MATERIAL; REPRODUCTION:

- DNA is the genetic material for all living organisms.
- Genes are segments of DNA that encode information critical for development. DNA is organized into structures called chromosomes.
- The genome is all the genetic information within an organism.
- Humans receive half their genetic information from each parent through the processes of replication, meiosis and fertilization.
- Relate the processes of cell division and emerging reproductive technologies to embryonic development.

Overview:
This 45 min lesson plan guides grade 9 students through the examination of two issues that have arisen from the understanding and utilization of the information from the Human Genome Project, namely personal genomics and human reproduction. The lesson explores how understanding and use of genetic information impacts many aspects of society (health, crime and justice, arts, environment, human history, human behavior). The lesson also highlights how it is currently possible to obtain genetic information, whether about specific genes, certain traits or the entire genome of both living people and embryos or unborn children.

Learning Objectives:
By the end of this lesson, students will be able to discuss key issues surrounding two controversies raised by human genomics: personal genomics and pre-implantation diagnosis.

Resources Needed:
Roll of masking tape
Projector and laptop
Worksheets (page 2 and 3 of this document)

Pre-Assessment: Create a value line in the classroom using a piece of masking tape across the floor. A “value line” ascertains students’ opinions in a quick and visual way by asking them to line up according to how strongly they agree or disagree with a statement or proposition[3].
Question 1: “ How often do you hear mention of DNA, genes, and genomics?” Label one end of the value line as “A lot,” the other as “Never”. Ask the students who stand closer to “A lot” where they have heard mention of these topics.
Question 2: “How big an impact do you think human genomics has on your life?” One extreme of the value line is “A big impact” the other “No impact”. Ask students to explain their positions.

Introduction:
Watch the video: Human Genome Project- Ethical, Legal, & Social Implications
http://www.youtube.com/watch?v=gkQJ26DAxfs

Activity: Personal Genomics
Hand out controversy worksheets to groups of 4-6 students. Read through the introductions together. Students work through scenario 1 individually, by writing down their answers. Students share their answers with their small groups. Ask for summaries of main findings from groups. Each small group then works through the second scenario. Bring the discussion back to the large group.

Activity: Human Reproduction
Watch the video: A natural selection.
http://www.youtube.com/watch?v=FqcA7jlqBN4
Have students in their small groups discuss the question on Human Reproduction Controversy worksheet. Discuss as large group.

Post- test/ Summary Have students stand on the value line according to their answers.
Question 1: "How big of an impact do you think human genomics has on your life?"
(A big impact- no impact)
Question 2: "How important is discussing and understanding the controversies around human genome sequencing? (Very important - Not important at all)

Key Concepts adapted from:
[1] B.C. Ministry of Education. Grade 9 Curriculum Package, online. www.bced.gov.bc.ca/irp [April 11, 2012]
[2] Dougherty M, Pleasants C, Solow L, Wong A, et al. (2011) A Comprehensive analysis of High School Genetics Standards: Are States Keeping Pace with Modern Genetics? CBE- Life Sciences Education (10): 318-327.
[3] Value Line definition from: http://www.humboldt.edu/celt/tips/value_line/

Credits: This lesson plan was developed by Jennifer McQueen, Jody Wright, and Joanne Fox as part of the science outreach efforts at the Michael Smith Laboratories at the University of British Columbia, http://bioteach.ubc.ca

Human Genomic Controversies:
Personal Genomics

We are now in a genomic age! The cost of sequencing genes and entire genomes is greatly decreasing. There are companies such as “23 and me”, that can do genetic testing for you. They can tell you your probability of developing certain diseases, or traits. Some of these include hair colour, lactose intolerance, and preference to working late at night. Something we still don’t know is how people will react to this type of information and how it will impact their lives and that of their families. Please read through the following situations and discuss as a group the questions.

Situation 1:
You submit a sample of saliva to 23 and me for genetic testing. You find out that you have an increased chance (5%) of developing Alzheimer's, which destroy memory functions of the brain. You also find out that you have a mutation in a gene that gives you resistance to many types of the stomach flu.

1. ***How do you feel about this information? (Are you confused, upset, curious, excited....)***
2. ***Who would you share this information with? (Your family, friends, a life partner....)***
3. ***Would this information change the way you lived?***

Situation 2:
A friend of yours has just had their genome sequenced and suggests you should too. It will cost about $1000 to have it done.

4. ***Would you have your full genome sequenced?***

You share 50% of your genetic material with each of your siblings and each of your parents. Many people today are making their genetic information publicly accessible. Making your information public can also help researchers study human DNA.

5. ***Should you require your family’s consent for this?***

6. ***What if you have an identical twin who shares 100% of your genetic sequence, should you require their consent before have a genetic test or making your genetic information public?***

Human Genomic Controversies:
Human Reproduction:

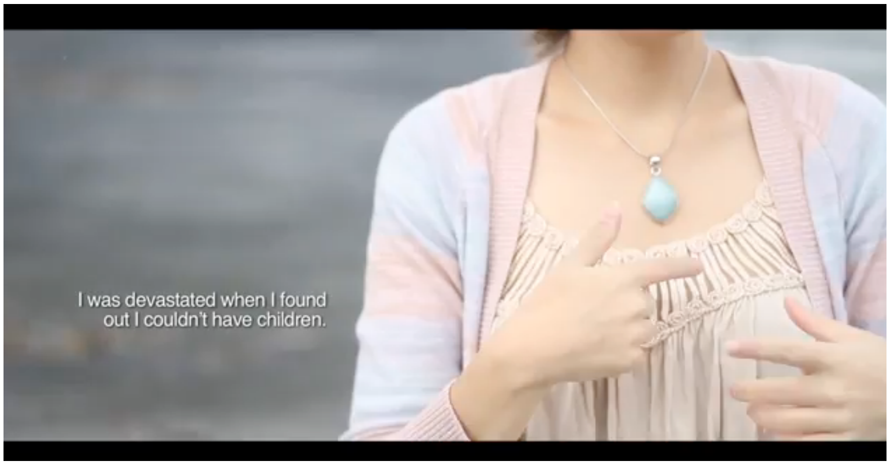

Canadians are choosing to have fewer children and at an older age. Many prospective parents are choosing to give their children the “best” possible chance they can by having their embryos screened for many genetic diseases and traits. Currently, many of the genetic diseases screened for are untreatable, effect patients early in life and have a known genetic basis. These can be disorders such as Hemophilia, a disorder that prevents patients from clotting blood, or Cystic Fibrosis that causes thick mucus to build up in the lungs and other parts of the body. As we increasingly understand the genetic causes of certain diseases or disorders, we can screen embryos for these as well. A recent example is the common breast cancer gene (*BRCA1* and *BRCA2*). Breast cancer occurs later in life and can be treatable, but patients can now screen for the genetic mutations in their embryos and choose to implant and carry to full term those embryos that do not have the mutations. While our understanding of the human genome has increased so to has the requirement to act responsibly with this information.

Please watch the accompanying video and answer the following questions.

- ***Should we decide which traits are screened for and which are not? How would you decide?***

Human Genomic Controversies:
Personal Genomics

Situation 1:

1) How do you feel about this information?

2) Who would you share this information with?

3) Would this information change the way you lived?

Human Genomic Controversies:
Human Reproduction

Should we decide which traits are screened for and which are not? How would you decide?

Terminology

Sequence: The order of DNA nucleotides.
Transcribe: Take genomic DNA and turn it into mRNA. The nucleotide T is replaced with U.
Translate: Taking mRNA and turning it into an amino acid sequence.

THANKS FOR COMING!!!!

MSL OUTREACH

GENOMICS

GRADE. 9 FIELD TRIP

Name: ______________________

http://www.bioteach.ubc.ca/

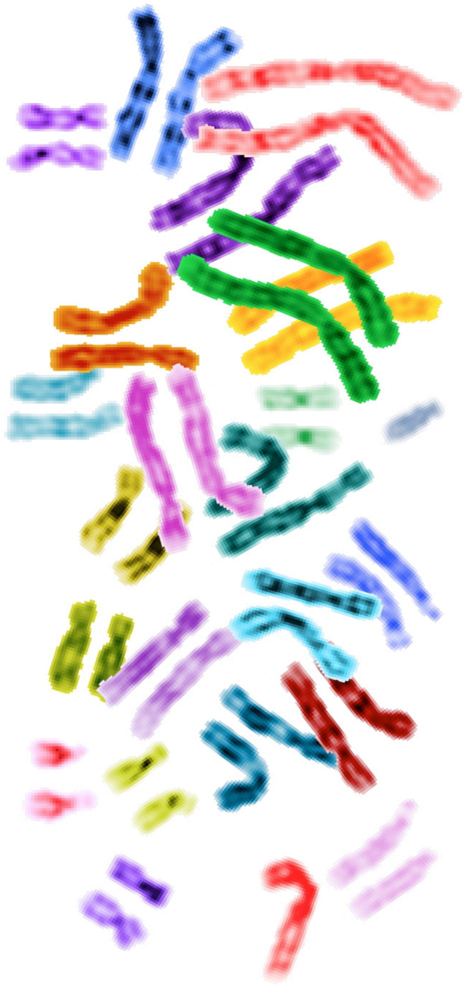

PHYSICAL AND CHEMICAL PROPERTIES OF DNA!

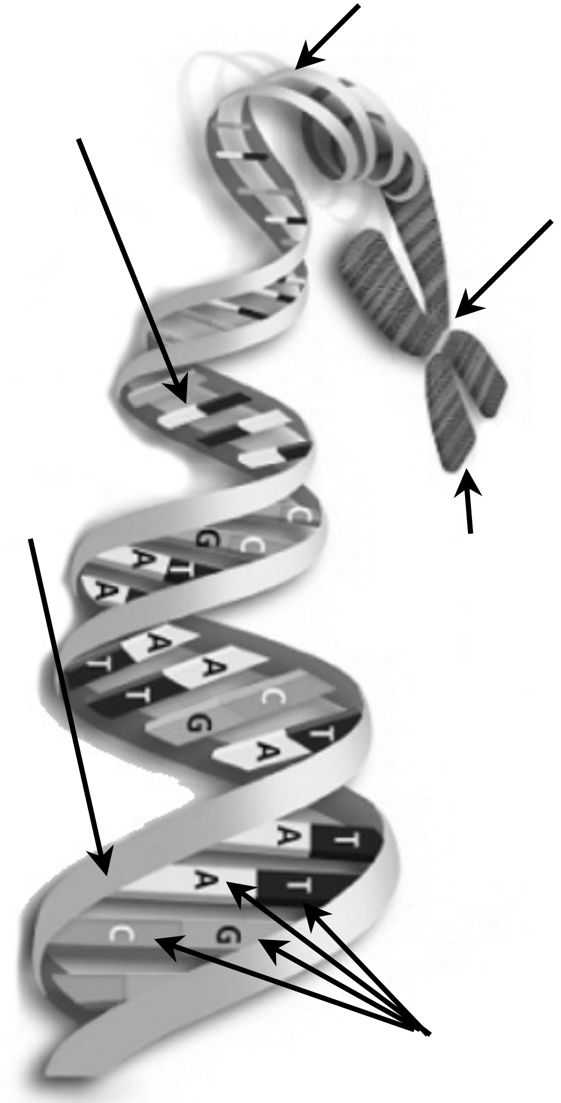

Name the ingredients used to extract DNA!

1. ______________________
2. ______________________
3. ______________________

THE INFORMATION IN DNA!

Write out your gene sequence.

Transcribe your sequence.

Using the table below translate your sequence.

Second base

First base	U	C	A	G	Third base
U	UUU, UUC – Phenyl-alanine F<br>UUA, UUG – Leucine L	UCU, UCC, UCA, UCG – Serine S	UAU, UAC – Tyrosine Y<br>UAA Stop codon<br>UAG Stop codon	UGU, UGC – Cysteine C<br>UGA Stop codon<br>UGG Tryptophan W	U C A G
C	CUU, CUC, CUA, CUG – Leucine L	CCU, CCC, CCA, CCG – Proline P	CAU, CAC – Histidine H<br>CAA, CAG – Glutamine Q	CGU, CGC, CGA, CGG – Arginine R	U C A G
A	AUU, AUC, AUA – Isoleucine I<br>AUG M Methionine start codon	ACU, ACC, ACA, ACG – Threonine T	AAU, AAC – Asparagine N<br>AAA, AAG – Lysine K	AGU, AGC – Serine S<br>AGA, AGG – Arginine R	U C A G
G	GUU, GUC, GUA, GUG – Valine V	GCU, GCC, GCA, GCG – Alanine A	GAU, GAC – Aspartic acid D<br>GAA, GAG – Glutamic acid E	GGU, GGC, GGA, GGG – Glycine G	U C A G

HUMAN GENOME PROJECT

What is a genome?

What is DNA sequencing?

Has the human genome been sequenced?

Answer Key for Sequence Alignment and Transcription /Translation

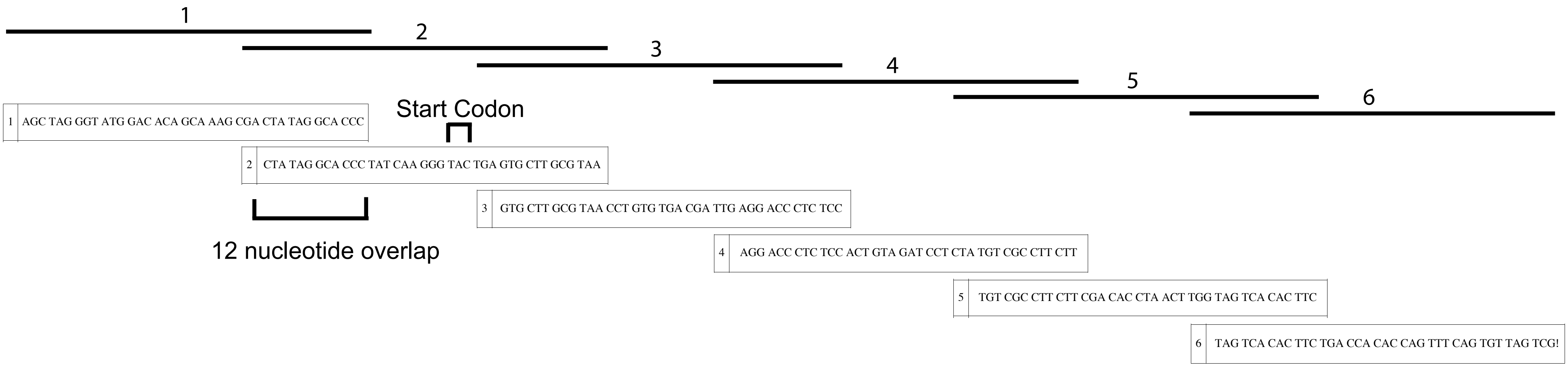

Steps in activity:

1) Students align the sequences with overlapping fragments. Their sequences should have the numbering cut off.

2) As a group find the start codon, by looking at the codon table and working backwards.

2) Tell the students that the sequence is 14 codons long after the start codon.

3) Have students write out the 14 codons that they will need to transcribe and translate. This can be done on the worksheet.

4) Students transcribe and translate the sequence by using the codon table.

5) The amino acid sequence is “THERIGHTANSWER”

	Codon#		1	2	3	4	5	6	7	8	9	10	11	12	13	14
Transcription	DNA	TAC	TGA	GTG	CTT	GCG	TAA	CCT	GTG	TGA	CGA	TTG	AGG	ACC	CTC	TCC
	RNA	AUG	ACU	CAC	GAA	CGC	AUU	GGA	CAC	ACU	GCU	AAC	UCC	UGG	GAG	AGG
Translation	A.A.	Start	T	H	E	R	I	G	H	T	A	N	S	W	E	R

PHYSICAL AND CHEMICAL PROPERTIES OF DNA!

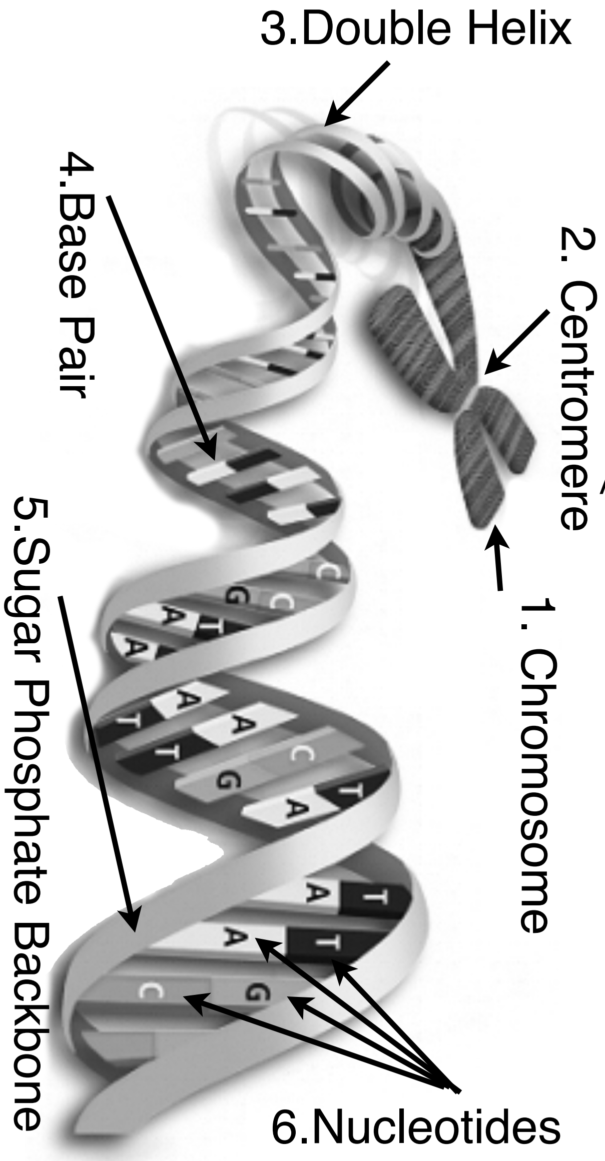

Name the ingredients used to extract DNA!

1. _Saline
2. _SDS (Soap)
3. _Ethanol

THE INFORMATION IN DNA!

Write out your gene sequence.

	1	2	3	4	5	6	7	8	9	10	11	12	13	14
TAC	TGA	GTG	CTT	GCG	TAA	CCT	GTG	TGA	CGA	TTG	AGG	ACC	CTC	TCC

Transcribe your sequence.

	1	2	3	4	5	6	7	8	9	10	11	12	13	14
AUG	ACU	CAC	GAA	CGC	AUU	GGA	CAC	ACU	GCU	AAC	UCC	UGG	GAG	AGG

Using the table below translate your sequence.

	1	2	3	4	5	6	7	8	9	10	11	12	13	14
M	T	H	E	R	I	G	H	T	A	N	S	W	E	R

Second base

First base	U	C	A	G	Third base
U	UUU, UUC Phenyl-alanine F<br>UUA, UUG Leucine L	UCU, UCC, UCA, UCG Serine S	UAU, UAC Tyrosine Y<br>UAA Stop codon<br>UAG Stop codon	UGU, UGC Cysteine C<br>UGA Stop codon<br>UGG Tryptophan W	U C A G
C	CUU, CUC, CUA, CUG Leucine L	CCU, CCC, CCA, CCG Proline P	CAU, CAC Histidine H<br>CAA, CAG Glutamine Q	CGU, CGC, CGA, CGG Arginine R	U C A G
A	AUU, AUC, AUA Isoleucine I<br>AUG Methionine M start codon	ACU, ACC, ACA, ACG Threonine T	AAU, AAC Asparagine N<br>AAA, AAG Lysine K	AGU, AGC Serine S<br>AGA, AGG Arginine R	U C A G
G	GUU, GUC, GUA, GUG Valine V	GCU, GCC, GCA, GCG Alanine A	GAU, GAC Aspartic acid D<br>GAA, GAG Glutamic acid E	GGU, GGC, GGA, GGG Glycine G	U C A G

HUMAN GENOME PROJECT

What is a genome?

All the DNA within an organism.

What is DNA sequencing?

Reading the order of nucleotides in a DNA molecule.

Has the human genome been sequenced?

Yes! Except for highly repetitive regions.

<u>Teacher Survey – MSL Grade 9 Genomics Field Trip*</u>

Date of Visit: ______________________

Teacher Name: ____________________

School Name: ______________________

How many students did you bring with you today? ______________

female students ______________

aboriginal students ______________

teacher participants ______________

1. Please list two highlights from today.

2. Please rate the following aspects from today's field trip. Please consider both the content and activities.

Section	Extremely Effective	Effective	Neutral	Not that Effective	Not Effective
Properties of DNA					
Information in DNA					
Genomics Controversies					
Jeopardy					

3. Use this space to expand on any of the aspects in Q2.

4. If you were to recommend this field trip program to your colleagues for next year what would you say?

5. Does this field trip program help you to deliver specific curricula, as outlined in the BC IRPs? Please expand on your thoughts here.

6. Do you have any suggestions for pre-trip or post-trip activities that would help integrate this field trip into your own classroom activities.

7. Please list any other suggestions for improving next year's field trip experience.

Thank you very much for your input. We commend you for your dedication to science teaching, education and outreach.

* This MSL outreach program is funded by the Natural Sciences and Engineering Research Council of Canada.

NSERC CRSNG

ambl

Michael Smith Labs Genomics Field Trip Program - Student Surveys

Student Surveys
* Required

Date of Visit (YYYY-MM-DD) *

Teacher Name *

School Name *

What's one new thing you learned today?

Please rate the following aspects of today's program

	Awesome	Good	Neutral	Not that Useful	Not Useful
Visit to AMBL lab space	○	○	○	○	○
DNA isolation experiment	○	○	○	○	○
DNA transcription / translation activity	○	○	○	○	○
Talking with the instructors	○	○	○	○	○
Learning about controversies in human genomics	○	○	○	○	○
Jeopardy game	○	○	○	○	○
Online research of genomics	○	○	○	○	○

If you were to recommend this field trip program to your fellow students for next year, what would you say?

After your visit to AMBL today, are you more interested in the Sciences and Engineering? *

- ◯ Way More Interested
- ◯ More Interested
- ◯ About the Same
- ◯ Less Interested
- ◯ Not Interested

Do you plan on pursuing your studies in the Sciences and Engineering? *

- ◯ Very Interested
- ◯ Interested
- ◯ Not Sure
- ◯ Not Interested

Are you considering a career in the Sciences and Engineering? *

- ◯ Very Interested
- ◯ Interested
- ◯ Not Sure
- ◯ Not Interested

Please list one suggestion for improving next year's field trip experience

Submit
